# Comparison of Maximum Signal Intensity of Contrast Agent on T1-Weighted Images Using Spin Echo, Fast Spin Echo and Inversion Recovery Sequences

**DOI:** 10.5812/iranjradiol.5452

**Published:** 2012-12-27

**Authors:** Mahmood Nazarpoor, Masoud Poureisa, Mohammad Hossein Daghighi

**Affiliations:** 1 Department of Radiology, Faculty of Paramedicine, Tabriz University of Medical Sciences, Tabriz, Iran; 2 Department of Radiology, Faculty of Medicine, Tabriz University of Medical Sciences, Tabriz, Iran

**Keywords:** Magnetic Resonance Imaging, Relaxation, Gadolinium DTPA

## Abstract

**Background:**

MRI is not able to directly measure the concentration of contrast agent. It is measured indirectly from the signal intensity (SI). It is very important to know how much contrast agent should be injected to receive a maximum SI in the region of interest (ROI).

**Objectives:**

The aim of this study was to investigate the maximum relationship between contrast concentration and signal intensity (SI) on T1-weighted images using spin echo (SE), fast spin echo (FSE) and inversion recovery (IR) sequences.

**Materials and Methods:**

To assess the relationship between SI and concentration, a water-filled phantom containing vials of different concentrations of gadolinium DTPA (Gd-DTPA) (0 to 19.77 mmol/L) or a constant concentration (1.2 mmol/L) of contrast agent was used. The vials of constant concentration were used to measure coil nonuniformity. The mean SI was obtained in the ROI using T1-weighted images. All studies were carried out using a 0.3 T clinical MR scanner with a standard head coil.

**Results:**

This study shows that maximum SI will appear at different ranges in different sequences. The maximum SI can be seen at concentrations of 5.95, 4.96 and 3.98mmol/L for SE, FSE and IR, respectively.

**Conclusion:**

Using standard imaging parameters, each MRI sequence reaches its maximum SI in a specific contrast concentration, which is highest in SE and least in IR in a comparison between SE, FSE and IR sequences.

## 1. Background

For improved contrast of MR images, paramagnetic-metal contrast agents are administered. While paramagnetic contrast medium passes through the tissue, it produces a local magnetic field inhomogeneity that leads to reduction in the transverse relaxation time (T2) and longitudinal relaxation time (T1) of the tissue. Decrease in T1 typically causes an increase in signal intensity (SI); whereas, decrease in T2 causes a decrease in SI (1). The T1-shortening effect is dominant at low concentrations of Gd-DTPA and the T2-shortening effect is dominant at high contrast concentrations and leads to a decrease in the SI. The net result of these effects on the MR signal intensity will depend on the image parameters and the type of imaging sequence (2, 3). The concentration of contrast agent in MRI is measured indirectly from SI. It is very important to know how much contrast agent should be injected to obtain a maximum SI in the region of interest (ROI). Increasing the concentration of contrast agent leads to a decrease in the SI; therefore, the obtained amount of optimal injected dose should be considered (1). Many studies have accepted the use of 0.1 mmol/kg of body weight injection in different T1-weighted sequences with different image parameters and MRI unit strength (4-10) Some other investigators performed different sequences with the use of high-dose contrast administration such as 0.2 mmol/kg of body weight (11, 12).

## 2. Objectives

The aim of this study was to investigate the concentration which leads to maximum signal intensity (SI) on T1-weighted images in spin-echo (SE), fast-spin-echo (FSE) and inversion recovery (IR) sequences with standard image parameters of the MR scanner.

## 3. Materials and Methods

### 3.1. Basics

Many factors such as the magnetic field strength, the pulse sequence parameters, the dose of the contrast agent, the injection rate and bolus volume, and the tissue topology may affect the SI (13-15). The MR sequence can influence the relationship between T1 and SI, which in turn is dependent on the contrast concentration (16). The relationship between SI and imaging parameters in the IR sequence can be written as:

**Equation 1:**
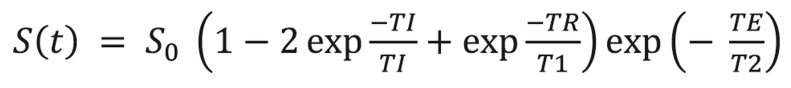


Where S(t) is the signal intensity after administration of the contrast agent, and S0 is the observed signal intensity when no magnetization preparation pre-pulses are applied or there is no contrast agent. TI and TR denote the inversion time and the repetition time, respectively. T1, TE, and T2 denote the tissue longitudinal relaxation time, the echo time and transverse relaxation time, respectively (3, 17, 18). Equation (1) with a concentration of contrast agent at time t (C (t)) can be described as (3):

**Equation 2:**
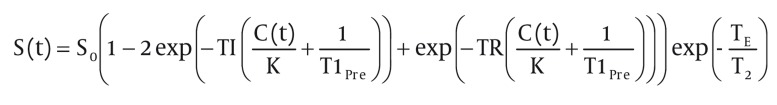


Where T1 _Pre_ is the longitudinal relaxation times before contrast application and K is a constant that depends on the contrast medium used (19). For saturation recovery (SE) sequences, the relationship between SI and image parameters and the concentration of contrast agent has been defined as:

**Equation 3:**
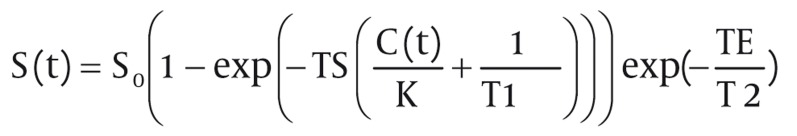


Where TS is the saturation time. At lower concentrations of the contrast agent, the term exp[-TE/T2] can be ignored in equations number 1, 2 and 3 (16, 18).

### 3.2. Calculation of Injection Volume of Contrast Agent

The amount of the injected dose required to establish a known concentration of the contrast agent in the ROI of the brain has been reported by Moody et al. (20):

**Equation 4:**
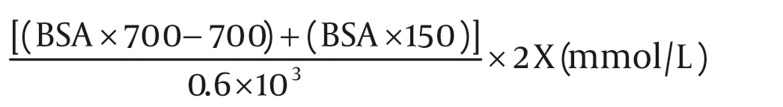


X is concentration of the contrast agent (mmol/L) in the ROI. BSA (m^2^) is the body surface area. BSA can be described as (21).

**Equation 5:**
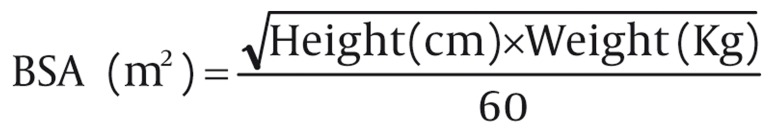


### 3.3. Phantom

**Figure 1:**
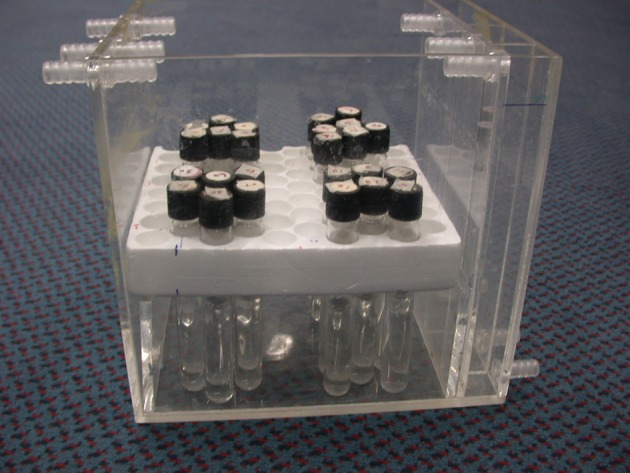
The phantom

**Figure 2:**
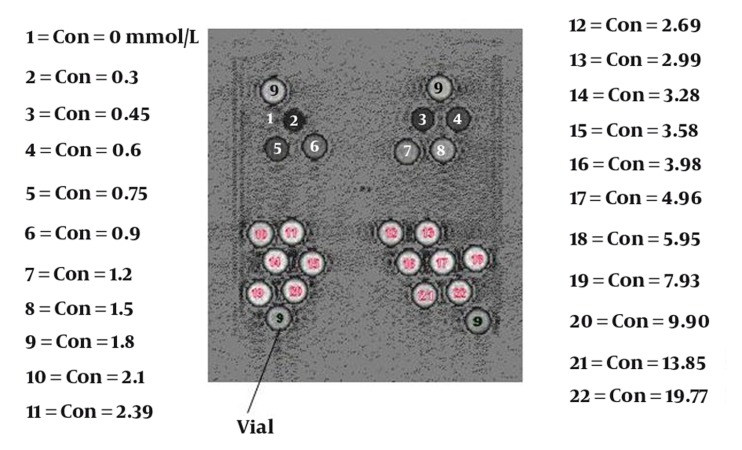
Coronal image of the phantom containing vials with different contrast concentrations

To find the relationship between the SI and concentration in different sequences, we designed a phantom to hold vials which contained either various or constant concentrations of the contrast agent (Figure 1). The vials of constant concentration (1.2 mmol/L) were used for measurement of coil non-uniformity (1, 3). Vials with different concentrations were used for measurement of the relationship between SI and concentration. A standard clinical head coil was used with the phantom. The vials were set vertically, and the axes of the vials were perpendicular to the image plane (Figure 2, coronal image). The phantom consisted of 25 vials containing 22 different contrast concentrations (glass tube, inner diameter approximately 15 mm filled with different concentrations of Gd-DTPA (Magnevist, Schering Health Care Ltd, West Sussex, UK). The concentration of Gd-DTPA ranged from 0 to 19.77 mmol/L (0.00, 0.30, 0.45, 0.60, 0.75, 0.90, 1.20, 1.50, 1.80, 2.10, 2.39, 2.69, 2.99, 3.28, 3.58, 3.98, 4.96, 5.95, 7.93, 9.90, 13.85 and 19.77 mmol/L).

Two experiments were performed, one using vials with different concentrations and one using vials with constant concentration. The vials in the phantom with a constant concentration were placed in exactly the same positions of the vials with different concentrations.

The non-uniformity of the coil was calculated from the SI of each vial with constant concentration and it was normalized to give a correction factor. For calculating the corrected SI for different concentrations, the SI of each vial was multiplied by its correction factor (22).

### 3.4. Image Acquisition

All studies were carried out with use of a 0.3 T clinical MR scanner (Hitachi Medical Corporation, AIRIS). The standard imaging parameters for constant and different concentrations were TR = 411 ms, TE = 15 ms, pixel size = 2×2 mm ^2^, matrix size =128×128 , slice thickness = 10 mm, flip angle = 90° for SE; TR = 816 ms, TE = 15 ms, pixel size = 2×2 mm ^2^, matrix size = 128×128, slice thickness = 10 mm, flip angle = 90°, Echo Train Length = 2 for FSE; and TR = 506 ms, TE = 15 ms, TI = 20 ms, pixel size = 2×2 mm ^2^, matrix size 128×128 slice thickness = 10 mm ^2^, flip angle = 90° for IR.

### 3.5. Image Analysis

After transferring the image data from the MR scanner to a personal computer, the image processing software Interactive Data Language (IDL, Research Systems, Inc. NY, USA; http://www.rsinc.com) was used for processing. Special programs were written to find the following:

- To measure the mean SI of the nine innermost pixels out of the total number of 44 pixels of each vial, to avoid partial volume effects.

- The correction factors of the non-uniformity of the coils from the SI of the vials with constant concentration. Then the SI of the vials with different concentration was multiplied by these factors to find the corrected SI.

- To draw the concentration versus the SI curve to measure the maximum value of the concentration of the contrast agent that gives a maximum SI.

These programs could be run from either a UNIX workstation or a personal computer.

## 4. Results

**Figure 3:**
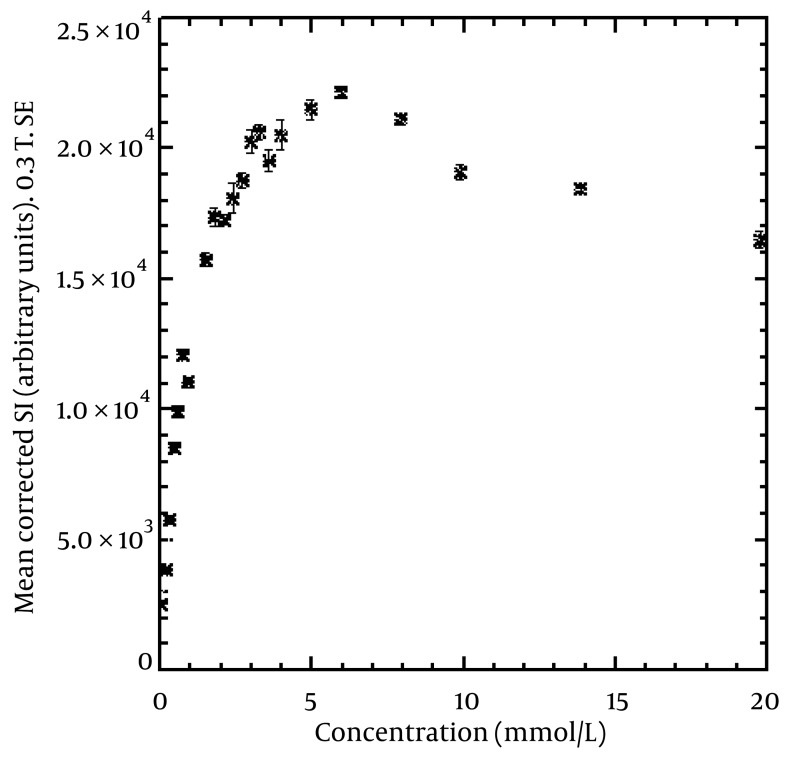
Mean corrected (for non-uniformity of the coil) SI from the nine innermost pixels of the vials versus concentration of contrast agent using SE. The maximum SI (22169) can be seen at a concentration of 5.95 mmol/L. The linear relationship between concentrations and corrected SI that gave an R^2^ of 0.95 was at 2.02 mmol/L. The error bars show the standard deviation of the SI in each vial.

**Figure 4:**
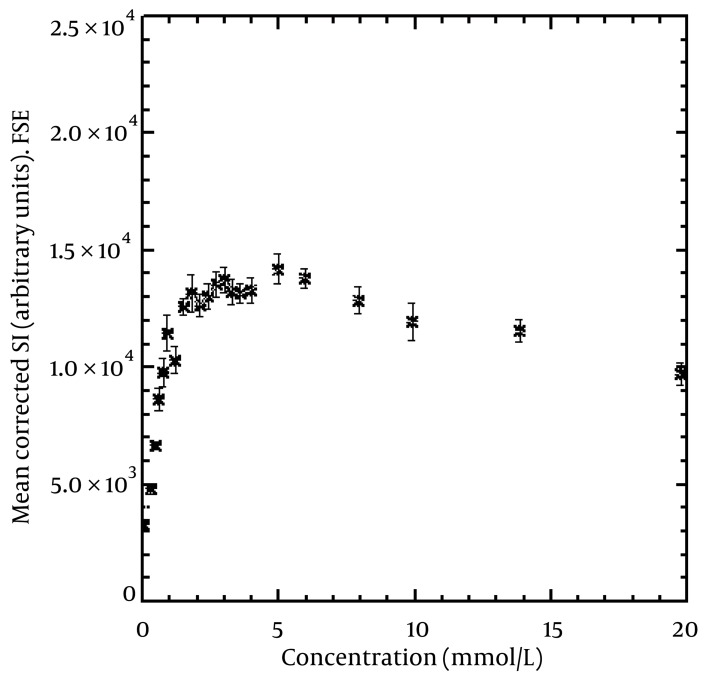
Mean corrected (for non-uniformity of the coil) SI from the nine innermost pixels of the vials versus concentration of contrast agent using FSE. The maximum SI (14218) can be seen at a concentration of 4.96 mmol/L. The figure illustrates that a linear relationship between SI and concentration reaches 1.18 mmol/L (R^2^ = 0.95). The error bars show the standard deviation of the SI in each vial.

**Figure 5:**
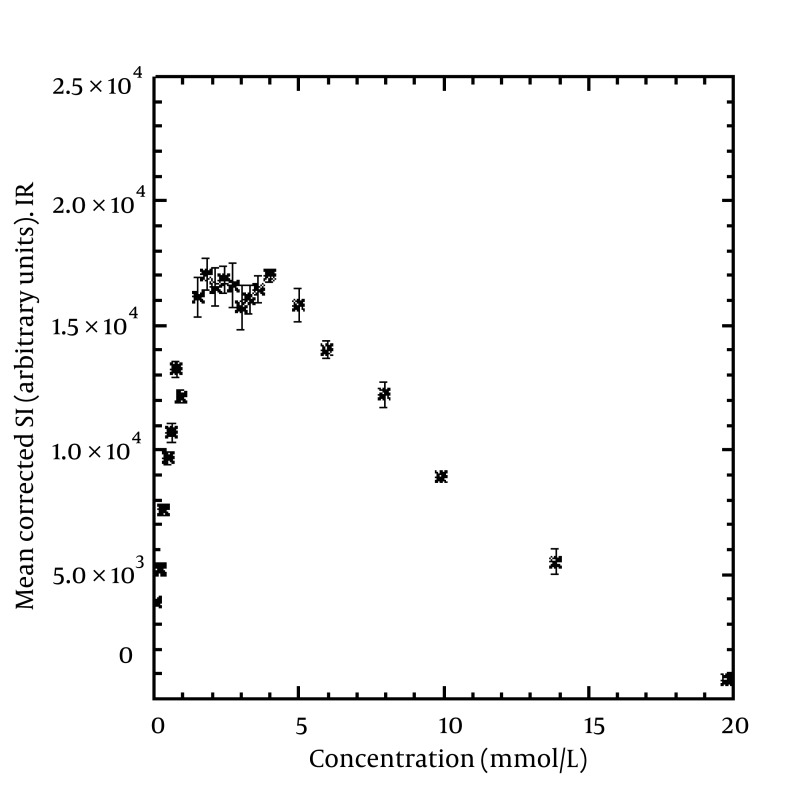
Mean corrected (for non-uniformity of the coil) SI from the nine innermost pixels of the vials versus concentration of contrast agent using IR. The maximum SI (17038) can be seen at a concentration of 3.98 mmol/L. The linear relationship between concentration and corrected SI resulting in an R^2^ of 0.95 was at 1.99 mmol/L. The error bars show the standard deviation of the SI in each vial.

Figures 3, 4 and 5 show the maximum relationship between SI and concentration in SE, FSE, and IR sequences. We detected maximum SI at concentrations of 5.95, 4.96 and 3.98 mmol/L for SE, FSE, and IR, respectively. The linear relationship between the concentrations and corrected SI that gave a 0.95 coefficient of determination (R^2^) was at 2.02, 1.18, and 1.99 mmol/L for SE, FSE, and IR, respectively. In addition, the figures show that at concentrations higher than the above, the SI will decrease. The error bars show the standard deviation of the SI from the nine innermost pixels in each vial. For example, 0.12, 0.10, and 0.08 mmol/kg of body weight (for an average body, i.e., height = 175 cm, weight = 85 kg) of contrast agent should be injected for SE, FSE, and IR sequences, respectively to give a maximum SI in the region of interest based on equation number 4. Higher doses of contrast will cause a decrease in SI.

## 5. Discussion

The concentration of a contrast agent such as Gd-DTPA in MRI has essentially no linear correlation with the SI. The contrast agent induces both T1- and T2- shortening effects, which lead to opposing effects on the SI. T1- shortening will increase the SI and T2- shortening will decrease it. As mentioned in equations number 2 and 3, both T1 and T2 may affect the SI at high concentrations. The T2-shortening effect becomes dominant at high concentrations and leads to a decreasing SI. The effect of T1-shortening is significant when the concentration of contrast agent is low (17-19). Melhem et al. (4) compared contrast-to-noise ratios between FSE (546/10 (TR/effective TE, echo-train length = 4) and SE (546/10, TR/TE) in T1-weighted MR sequences of 32 enhancing brain lesions. MR images were obtained at 1.5 T after IV administration of 0.10 mmol/kg gadopentetate dimeglumine. Sugahara et al. (5) evaluated the T1-FSE sequence (690/12 (TR/TE _effective_); echo train length = 3) to determine whether this technique can replace the conventional T1-weighted spin-echo sequence (690/14 (TR/TE)) for routine contrast-enhanced imaging. They obtained the images after intravenous administration of 0.1 mmol/kg gadopentetate dimeglumine with a 1.5 T magnet.

Qian et al. (6) used T1 IR and T1 SE sequences for detecting brain metastases. For contrast enhancement, they administered gadopentetate dimeglumine (Magnevist) with the standard dose of 0.1 mmol/kg of body weight. Kizildağ et al. (7) used T1-weighted (T1W), FSE T2-weighted (FSE T2W) and fluid-attenuated inversion recovery (FLAIR) sequences to assess the features of normal brain development in terms of myelination in infants and young children on a 1 Tesla MR unit with administration of 0.1 mg/Kg of Dormicum. Zhou et al. (8) injected 0.1 mmol/kg of body weight of contrast agent (Gd-DTPA) to evaluate intracranial tumors by SE (TR/TE = 440/14 ms) T1-weighted images, using a 1.5 MRI system.

Tomura et al. (9) used SE ((TR/TE) (300-540/8-14 ms, 1.5 T) and FSE FLAIR sequences to compare brain tumors by injecting 0.1 mmol/Kg of body weight of gadopentetate dimeglumine.

Alibek et al. (10) performed SE ((TR/TE) (470/17 ms, 1.5 T) and FLAIR (TI/TR (860/1720 ms)) sequences by injecting 0.1 mmol/Kg body weight of gadolinium contrast agent to detect brain lesions in an unsedated pediatric patient with a 1.5 T MRI system. Al-Saeed et al. (11) performed FLAIR (relaxation/repetition time/time interval (TI)/number of excitations, 1920/8.2/750/2) and FSE (relaxation/repetition time/echo time/number of excitations, 600/8.1/2) T1-weighted sequences for comparison of 20 patients with brain lesions. They administered 0.2 mmol/Kg of gadolinium chelate for each patient and used a 1.5 T MRI system. Kakeda et al. (12) compared SE (TR/TE = 520/9 ms), IR-FSE (TR/TE/TI = 2500/9.1/1000 ms, echo train length = 7), and 3D-gradient echo (GRE) sequences to detect brain metastases at 3 T scanner. They administered 0.2 mmol/kg of body weight gadoteridol for all patients. They reported that a 0.2 mmol/L of contrast agent concentration gave the maximum SI in SE and IR-FSE sequences in a phantom study using a 3 T MRI system. Our study showed that 5.95, 4.96, and 3.98 mmol/L of contrast agent concentration for SE, FSE, and IR sequences, respectively, gave a maximum SI in the region of interest. The difference between the results of this study and that of Kakeda et al. (12) may be due to differences in the use of different TR, TE, and MRI strengths. Different image parameters and MRI strengths can have an effect on the maximum relationship between SI and contrast agent concentration (1, 13, 14). Many studies have been performed without considering imaging parameters, imaging sequences and strength of the MRI system, with injection of 0.1 or 0.2 mmol/kg of body weight of contrast agent. Our study showed that the maximum concentration which leads to maximum SI depends on the image sequence. At higher doses as mentioned in the results, there was not only an increased SI, but also a decreased SI (Figures 3, 4 and 5).

This study shows that maximum SI will appear at different concentrations when different sequences are used with standard imaging parameters. A maximum SI can be obtained at high concentrations using the SE sequence, but not FSE and IR sequences. In addition, a maximum SI can be seen at higher concentrations for the FSE sequence and not in IR sequences. The results of this study can be used in clinical studies in the future.
